# Reattribution to Mind-Brain Processes and Recovery From Chronic Back Pain: A Secondary Analysis of a Randomized Clinical Trial

**DOI:** 10.1001/jamanetworkopen.2023.33846

**Published:** 2023-09-28

**Authors:** Yoni K. Ashar, Mark A. Lumley, Roy H. Perlis, Conor Liston, Faith M. Gunning, Tor D. Wager

**Affiliations:** 1 Division of Internal Medicine, Department of Medicine, University of Colorado Anschutz Medical Campus, Aurora; 2 Department of Psychology, Wayne State University, Detroit, Michigan; 3 Center for Quantitative Health, Massachusetts General Hospital and Harvard Medical School, Boston, Massachusetts; 4 Department of Psychiatry, Weill Cornell Medical College, New York, New York; 5 Department of Psychological and Brain Sciences, Dartmouth College, Hanover, New Hampshire

## Abstract

**Importance:**

In primary chronic back pain (CBP), the belief that pain indicates tissue damage is both inaccurate and unhelpful. Reattributing pain to mind or brain processes may support recovery.

**Objectives:**

To test whether the reattribution of pain to mind or brain processes was associated with pain relief in pain reprocessing therapy (PRT) and to validate natural language–based tools for measuring patients’ symptom attributions.

**Design, Setting, and Participants:**

This secondary analysis of clinical trial data analyzed natural language data from patients with primary CBP randomized to PRT, placebo injection control, or usual care control groups and treated in a US university research setting. Eligible participants were adults aged 21 to 70 years with CBP recruited from the community. Enrollment extended from 2017 to 2018, with the current analyses conducted from 2020 to 2022.

**Interventions:**

PRT included cognitive, behavioral, and somatic techniques to support reattributing pain to nondangerous, reversible mind or brain causes. Subcutaneous placebo injection and usual care were hypothesized not to affect pain attributions.

**Main Outcomes and Measures:**

At pretreatment and posttreatment, participants listed their top 3 perceived causes of pain in their own words (eg, football injury, bad posture, stress); pain intensity was measured as last-week average pain (0 to 10 rating, with 0 indicating no pain and 10 indicating greatest pain). The number of attributions categorized by masked coders as reflecting mind or brain processes were summed to yield mind-brain attribution scores (range, 0-3). An automated scoring algorithm was developed and benchmarked against human coder–derived scores. A data-driven natural language processing (NLP) algorithm identified the dimensional structure of pain attributions.

**Results:**

We enrolled 151 adults (81 female [54%], 134 White [89%], mean [SD] age, 41.1 [15.6] years) reporting moderate severity CBP (mean [SD] intensity, 4.10 [1.26]; mean [SD] duration, 10.0 [8.9] years). At pretreatment, 41 attributions (10%) were categorized as mind- or brain-related across intervention conditions. PRT led to significant increases in mind- or brain-related attributions, with 71 posttreatment attributions (51%) in the PRT condition categorized as mind- or brain-related, as compared with 22 (8%) in control conditions (mind-brain attribution scores: PRT vs placebo, *g* = 1.95 [95% CI, 1.45-2.47]; PRT vs usual care, *g* = 2.06 [95% CI, 1.57-2.60]). Consistent with hypothesized PRT mechanisms, increases in mind-brain attribution score were associated with reductions in pain intensity at posttreatment (standardized β = −0.25; *t*_127_ = −2.06; *P* = .04) and mediated the effects of PRT vs control on 1-year follow-up pain intensity (β = −0.35 [95% CI, −0.07 to −0.63]; *P* = .05). The automated word-counting algorithm and human coder-derived scores achieved moderate and substantial agreement at pretreatment and posttreatment (Cohen κ = 0.42 and 0.68, respectively). The data-driven NLP algorithm identified a principal dimension of mind and brain vs biomechanical attributions, converging with hypothesis-driven analyses.

**Conclusions and Relevance:**

In this secondary analysis of a randomized trial, PRT increased attribution of primary CBP to mind- or brain-related causes. Increased mind-brain attribution was associated with reductions in pain intensity.

## Key Points

**Question:**

Does pain reprocessing therapy, a promising psychological treatment, help patients view primary chronic pain as caused by mind or brain processes?

**Findings:**

In this secondary analysis of clinical trial data, natural language methods were applied to understand patients’ beliefs about the underlying causes of their primary chronic back pain. Pain reprocessing therapy led to significant increases in mind- or brain-related attributed causes of pain and increases in mind-brain attributions were associated with reduced pain.

**Meaning:**

These results suggest that patients’ pain attributions are often inaccurate, and that promoting mind- or brain-related attributions may support the effective treatment of primary chronic pain; helping patients to consider pain as “in the brain” may help relieve it.

## Teaser

This secondary analysis of a randomized clinical trial examines whether patient reattribution of primary chronic back pain to brain- or mind-related causes is associated with pain relief in pain reprocessing therapy.

## Introduction

Beliefs that pain is due to peripheral pathophysiology (eg, a bulging disc, osteoarthritis) are common. Yet, peripheral findings are often incidental in nature and not the predominant cause of symptoms. For patients with primary or nociplastic chronic pain—including the majority of cases of chronic back pain, tension headache, and many other pain conditions—pain is driven predominantly by central upregulation and threat learning processes.^1,2,3^ For these patients, the inaccurate belief that pain signifies tissue damage may promote fear, avoidance, disuse, and the persistence of pain.^4,5^

We recently developed pain reprocessing therapy (PRT), a novel psychological treatment aiming to help patients reframe primary chronic pain as caused by nondangerous, reversible brain pathways. PRT presents primary pain as what could be described as a “false alarm” of tissue damage that can be reversed. PRT demonstrated promising efficacy in a 2022 clinical trial^6^ of 151 adults with low-moderate severity chronic back pain: 66% of participants randomized to PRT were pain-free or nearly so at posttreatment, as compared with fewer than 20% of placebo and usual care controls. Better understanding the psychological mechanisms of PRT is critical.

In medically unexplained symptom disorders, the misattribution of symptoms to bodily damage or disease is recognized as a central factor driving dysfunction.^7,8,9,10^ Patients’ symptom attributions have rarely been investigated in chronic pain, although extant work suggests that attributions center on peripheral tissue pathology.^11,12^ This is understandable: imaging studies often reveal incidental findings (eg, small disc bulges) that can be easily misinterpreted as causal of pain, and pain is naturally associated with injury, rendering other attributions unintuitive.^13,14^ We hypothesized that the reattribution of pain to mind- or brain-related processes: (1) occurs in PRT, and (2) is associated with pain reduction.

We measured pain attributions before and after treatment using open-ended, free-text responses asking participants to describe the perceived causes of pain in their own words. Natural language approaches complement other valuable measurement tools. Relative to multiple choice–format questions, they provide a minimally constrained approach to studying how patients spontaneously think, capturing a broader set of concepts and beliefs than otherwise possible. Relative to qualitative analyses, they are quantitative and scalable (easily applied to large text data sets, eg, social media, electronic health record data). Natural language methods have been valuable in several psychiatric applications, including dimensional phenotyping^15^ and prediction of treatment response,^16^ but we are not aware of previous applications to symptom attributions.

## Methods

The pain attribution data presented here were collected as part of a preregistered clinical trial (NCT03294148) conducted from 2017 to 2019, with the current analyses conducted from 2020 to 2022. Primary outcomes have been previously reported,^6^ but not the attribution data. We provide a brief overview of the trial design here, with full details available in the prior publication and online.^6,17^ This manuscript follows the Consolidated Standards of Reporting Trials (CONSORT) reporting guideline.

### Participants

Participants aged 21 to 70 years with back pain for at least half the days of the last 6 months and 1-week pain intensity averaging 4 or higher on the 10-point Brief Pain Inventory were recruited from the Boulder, Colorado, area via printed and digital advertisements. We targeted primary (nociplastic) chronic back pain (CBP), excluding patients with leg pain worse than back pain, a history of metastasizing cancer, pain-related compensation or litigation, or severe mental illness. Participants provided written informed consent as approved by the University of Colorado institutional review board. Race (including American Indian or Alaskan Native, Asian and Pacific Islander, Black, White, and other or unknown), ethnicity, and gender were self-reported by participants; sex assigned at birth was not collected. Participants were randomized to PRT, placebo, or usual care, with equal probability using an imbalance minimization algorithm. No major changes to study protocol occurred after trial commencement, and sample size was determined by power analyses (eMethods in Supplement 2).

### Interventions

In the PRT group, participants completed a 1-hour telehealth session with a physician followed by 8 individual 1-hour sessions with a therapist twice weekly for 4 weeks. Treatment assessed centralized vs peripheral contributions to pain and provided education on mind and brain generators of chronic pain, substantiated by personalized supporting evidence (eg, spatial spread of symptoms, history of multiple somatic symptoms).^18^ Treatment aimed to shift pain attributions using guided somatically focused reappraisal exercises and by promoting insight into links between emotional or psychological states and pain. A further description of PRT is provided in the eMethods in Supplement 2.

Participants in the open-label placebo group watched 2 videos^19^ describing how placebos can powerfully relieve pain even when known to be inert and received a subcutaneous injection openly described as saline into the back during an empathic, validating clinical encounter in an orthopedic medical center. This intervention did not directly target pain attributions. Participants in the usual care group agreed to continue current care as usual and not start new treatments during study participation.

### Measures

Participants completed self-report measures at baseline (prerandomization) and posttreatment using an electronic database (REDCap) and masked research assistants. Attributions were collected using an adapted form of the final item of the Illness Perceptions Questionnaire,^20^ instructing participants to “please list in rank-order the 3 most important factors that you believe caused your pain” in a short-answer format. Pain intensity was measured as last-week average pain (0 to 10 numerical rating, with 0 indicating no pain and 10 indicating greatest pain), using the first item of the Brief Pain Inventory Short Form.^21^ Questionnaire measures of pain beliefs included: (1) the Tampa Scale of Kinesiophobia (TSK-11),^22^ which has a 2-factor structure measuring activity avoidance and harm beliefs^23,24^; (2) the Pain Catastrophizing Scale (PCS),^25^ assessing pain-related amplification, rumination, and helplessness; and (3) the Survey of Pain Attitudes (SOPA) 2-item Emotions subscale,^26^ assessing perceived influences of stress and emotion on pain.

### Analyses

We conducted 4 sets of analyses of the free-text pain attributions: (1) categorization of the attributions by human coders, with the total number of attributions assigned to a category reflecting mind or brain processes quantified as mind-brain attribution scores; (2) computing the frequencies of specific words used in attributions; (3) application of a data-driven (unsupervised) text scaling algorithm identifying the principle semantic dimensions underlying the attributions data; and (4) developing a scalable, automated algorithm scoring attributions for mind- and brain-related concepts.

#### Human Coder–Derived Categorization

The authors reviewed the free-text attributions while masked to treatment condition and time point and developed conceptually coherent categories of attributions based on this review of the data. Two masked authors then assigned each participant-generated attribution to a category, with disagreement resolved by discussion. Three categories were considered by the authors as mind- or brain-related. We tallied how many of the 3 attributions provided by each participant were assigned to one of these categories, yielding a mind-brain attribution score for each participant at each time point ranging from 0 (no mind- or brain-related attributions) to 3 (all attributions mind- or brain-related).

Using these mind-brain attribution scores, we tested for (1) their association with questionnaire measures of pain beliefs (TSK-11, PCS, SOPA-emotions) and demographic attributes at baseline, (2) effects of PRT vs control conditions to measure target engagement, and (3) for associations with changes in pain intensity, harm beliefs, or activity avoidance in PRT, investigating whether reattribution might be a psychological mechanism of PRT. We additionally investigated longer-term effects of reattribution, examining associations between changes in mind-brain attribution scores and pain intensity at 1-year follow-up. Finally, we conducted a longitudinal mediation analysis testing whether the effects of PRT vs combined controls on 1-year follow-up pain intensity was mediated by pre-to-posttreatment changes in mind-brain attribution scores. Statistical model details are provided in the eMethods in Supplement 2.

#### Word Frequency Changes

We identified the specific words with the largest pre-to-posttreatment changes in frequency within the PRT condition. The word counts reflect how often participants used particular words in their attributions. This complemented the human coder–derived categorizations in 2 ways: it provided an objective outcome (not based on human coder decisions), and it provided a finer-grained outcome relative to the coarser categories.

#### Text Scaling

Text-scaling algorithms characterize the semantic structure of collections of documents, identifying principal dimensions based on patterns of word cooccurrence. We used an algorithm including regularization methods that provides enhanced reliability for short documents.^27,28^ Text scaling is commonly used to identify ideological dimensions underlying political texts (eg, political left vs right); we hypothesized that a text-scaling algorithm might identify a mind-brain vs structural-biomechanical dimension underlying pain attributions and that post-PRT participants would be further toward the mind-brain end of such a dimension (eMethods in Supplement 2).

#### Automated Attribution Scoring Algorithm

We sought to develop an automated, scalable method for scoring whether attributions were mind- or brain-related. Five expert clinicians who had not seen the participant-provided attributions generated words that they would consider mind- or brain-related, which we preprocessed using standard methods (eMethods in Supplement 2). A word-counting algorithm computed whether each attribution contained words from the expert-derived list (yes-no scoring), yielding an algorithmically derived mind-brain attribution score ranging from 0 (no attributions contained expert-derived mind or brain words) to 3 (all 3 attributions contained expert-derived mind or brain words). We benchmarked the performance of the automated word-counting algorithm relative to the human coder–derived mind-brain attribution scores using Cohen κ (eMethods in Supplement 2). In contrast to text scaling, the automated algorithm was trained independently of the data and provided scores on the same scale as the manual coding approach, enabling direct comparison and validation.

## Results

**Figure 1.**
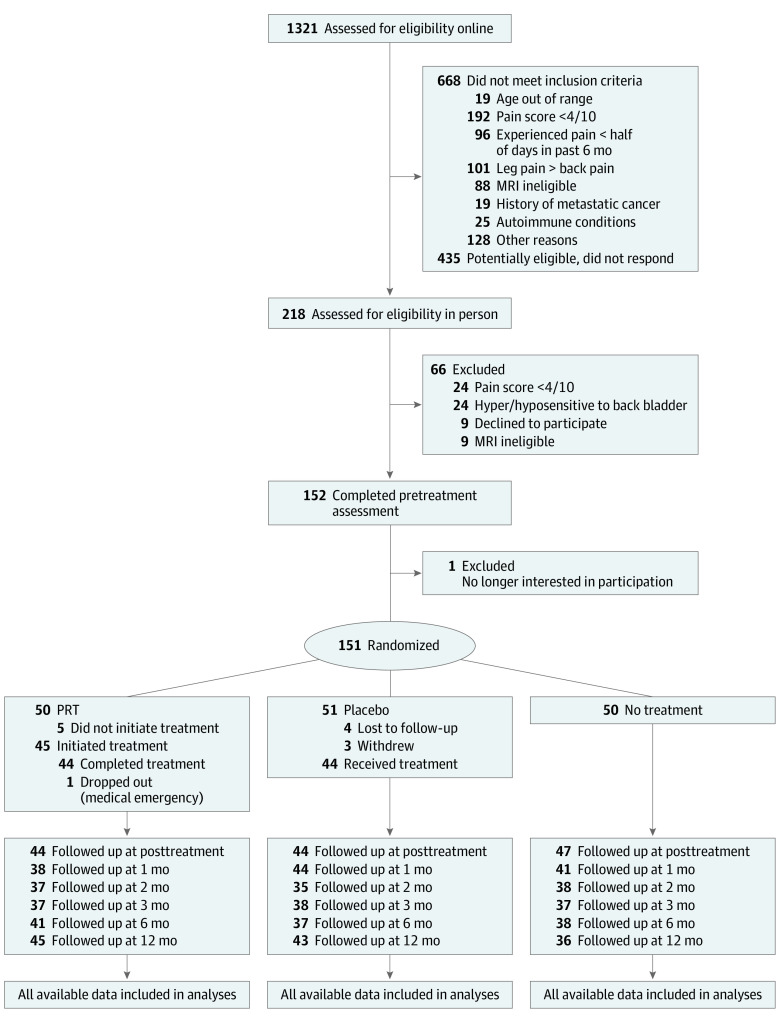
Study Flow Diagram

A total of 151 participants were randomized and provided pain attributions at pretreatment, and 135 (89.4%) completed their assigned treatment condition and provided attributions at posttreatment (mean [SD] age, 41.1 [15.6] years; 81 female [54%]; 134 White [89%]) (Figure 1). No adverse events were reported. The sample had moderate pain intensity (mean [SD] score, 4.10 [1.26]) and disability (mean [SD] Oswestry Disability Index, 23.34 [10.17]) at pretreatment, with mean (SD) CBP duration of 10.0 (8.9) years. Preexisting spinal imaging was available in 20 patients in the PRT condition, all of whom had at least 1 spinal anomaly, with a median of 4 anomalies per participant.^6^ Full sample demographics are available in Ashar et al.^6^

All participants provided 3 substantive or meaningful pain attributions, except for 1 participant (who wrote “???”). Attributions ranged in length from 1 to 39 words, with a mean (SD) of 3.11 (3.26) words. Of 891 attributions coded, only 38 (4%) were categorized discrepantly between coders.

### Human Coder–Derived Categorization

**Figure 2.**
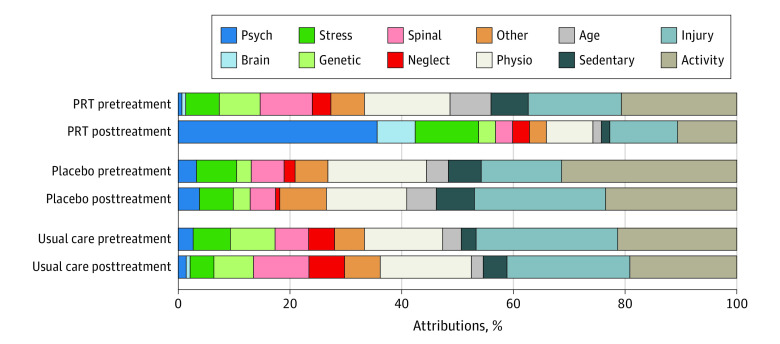
Pain Attribution Category Prevalence Pain attributions were assigned to categories by coders masked to treatment condition and time point. Attribution category prevalence rates for each condition at each time point are shown, with each participant contributing 3 attributions. Substantial increases in psychological and brain attributions (psychological, brain, and stress categories) were observed in the PRT condition, with relatively little attribution changes in placebo or usual care. Numeric values for attribution counts are shown in eTable 1 in Supplement 2. Definitions and exemplars for each category are presented in the Table.

Participants’ pain attributions varied widely (word cloud presented in eFigure in Supplement 2). We grouped them into 11 conceptually coherent categories (Table, Figure 2). The most prevalent attribution categories at pretreatment were activity (111 attributions [25%]), injury (85 attributions [19%]), and physiological (71 attributions [16%]). Three categories were considered by the authors as mind- or brain-related: stress (30 [7%]), psychological (10 [2%]), and brain (1 [0.2%]), all of which were low prevalence at pretreatment.

**Table.** Categories of Patient Attributions Regarding Perceived Causes of Pain

Category^a^	Description	Example attributions
Spinal condition	Spinal injury or anatomical issue	“Degenerative processes” “Scoliosis” “Overworking my back muscles to cause spinal disc to bulge”
Physiological	Peripheral soft tissue, potential for behavioral remediation	“Lack of flexibility” “Weak core and abdominal muscles” “Bad posture”
Injury	Reference to a specific past injury event	“Sports injury” “Falling—ice skating as a child” “Pushing a car out of the snow 40 years ago”
Activity	General engagement in an activity (no reference to a specific injury event)	“Gymnastics” “Rowing” “Playing three sports in high school”
Neglect	Failure to engage in care or treatment	“Lack of treatment early in life” “Inadequate self-care” “Too busy to take care of my body properly”
Sedentariness	Inactivity, sedentary lifestyle, prolonged sitting	“Sedentary work/lifestyle” “Inactive lifestyle—I sit a lot in front of a computer or in a car” “Sitting for 8-14 h/day”
Stress	Attribution focuses on “stress,” with little elaboration	“Stress” “Stressful job”
Psychological	A psychological attribution besides stress. Generally related to negative affect.	“Personality” “Outlook on life” “Guilt” “Putting everyone else’s needs before my own”
Brain	Neurobiological or pain-processing related attribution	“Neural pathways” “Brain pathways that developed and stayed even after healing” “Overactive pain response”
Hereditary or congenital	Hereditary or congenital attribution	“Genetics” “Birth defect” “Hereditary”
Age	Aging-related attribution	“Age” “Aging”

^a^ Categories were derived from discussion among authors masked to treatment condition and time point analyses. Prevalence rates for each category are shown in Figure 2 and eTable 1 in Supplement 2.

Each participant’s mind-brain attribution score was computed as the number of attributions assigned by the human coders to a mind- or brain-related category (stress, psychological, and brain). Mind-brain attribution scores ranged from 0 to 3 and were low at baseline (mean, 0.27; median, 0), which indicated predominantly non–mind or brain attributions (Figure 2).

At baseline, mind and brain attribution scores were positively correlated with a stronger perceived influence of stress and emotion on pain (SOPA Emotion subscale) (*r*_149_ = 0.22; *P* = .007), were positively correlated with pain intensity (*r*_149_ = 0.17; *P* = .03), and were marginally higher for women than men (*d* = 0.28; *P* = .07). Baseline mind-brain attribution scores were not correlated with harm beliefs or activity avoidance (TSK-11), pain catastrophizing (PCS), age, or duration of back pain.

**Figure 3.**
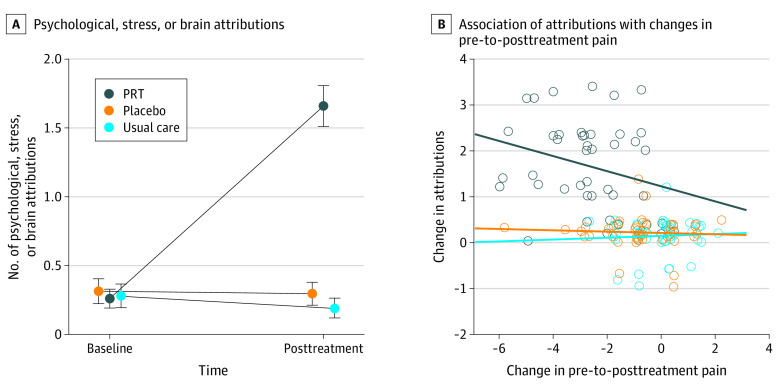
Effects of Pain Reprocessing Therapy (PRT) on Patients’ Attributions Regarding the Underlying Causes of Chronic Back Pain Mind-brain attribution scores were computed by counting how many of the 3 attributions provided by participants were categorized as psychological, stress, or brain by coders masked to treatment condition and time point (range: 0-3, with higher scores indicating more mind or brain attributions). A, PRT produced large pre-to-posttreatment increases in mind-brain attribution scores relative to placebo and usual care. B, Within the PRT condition, pre-to-posttreatment increases in mind and brain attributions were significantly associated with decreases in pain intensity.

PRT led to substantial increases in mind-brain attributions (Figure 3A). Mind-brain attribution scores increased for PRT vs placebo (β = 1.57, *t*_130_ = 10.83; *P* < .001) corresponding to *g* = 1.95 (95% CI, 1.45-2.47), and for PRT vs usual care (β = 1.64, *t*_130_ = 11.65; *P* < .001) corresponding to *g* = 2.06 (95% CI, 1.57-2.60).

Increases in mind-brain attribution scores were associated with decreases in pain intensity at posttreatment in the PRT condition (standardized β = −0.25, *t*_127_ = −2.06; *P* = .04), consistent with hypotheses (Figure 3B); interactions for condition × change in mind-brain attribution score on pain intensity were not significant. Examining simple correlations, pre-to-posttreatment changes in mind-brain attribution scores and pain intensity were *r*_133_ = −0.52 (*P* < .001) across the full sample and *r*_42_ = −0.28 (*P* = .06) within the PRT condition, corresponding to roughly 9% of variance explained by changes in mind-brain attribution scores within the PRT condition. These effects were largely maintained when examining pain intensity at 1-year follow-up, with standardized β = −0.33 (*t*_108_ = −2.42; *P* = .02) and simple correlations of *r*_114_ = −0.44 (*P* < .001) across the full sample and *r*_34_ = −0.25 (*P* > .99) within the PRT condition. Changes in mind-brain attribution scores partially mediated the effects of PRT vs control on pain intensity at 1-year follow-up (standardized β = −0.35 [95% CI, −0.07 to −0.63]; *P* = .05).

Increases in mind-brain attribution scores were associated with decreased harm beliefs and activity avoidance (TSK-11) at posttreatment in the PRT condition, standardized β = −0.27 (*t*_127_ = −2.41; *P* = .02), consistent with hypotheses; interactions for condition × change in mind-brain attribution scores interactions were not significant. Pre-to-posttreatment changes in mind-brain attribution scores and pre-to-post changes in harm beliefs or activity avoidance were correlated *r*_133_ = −0.57 (*P* < .001) in the full sample and *r*_42_ = −0.37 (*P* = .01) within the PRT condition. Increases in mind-brain attribution scores were similarly associated with decreased catastrophizing (eResults in Supplement 2).

#### Word Frequency Changes

The word with the largest increase in prevalence in the PRT condition was *anxiety*. Several emotion-related words (eg, *fear*, *feelings*, *emotion*, *people*) and neurobiological words (*neural*, *pathways*) were absent at baseline but present in PRT participant attributions at posttreatment, reflecting the introduction of a novel vocabulary. PRT participants decreased their use of words reflecting biomedical attributions, including *activity*, *weight*, *disc*, and *sport* (eTable 2 in Supplement 2).

#### Text Scaling

**Figure 4.**
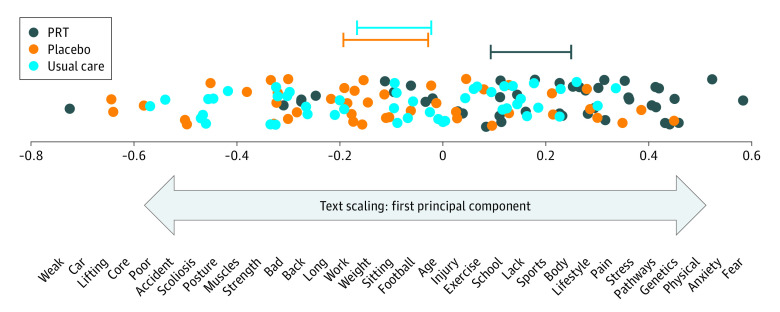
Scaling Analysis of Posttreatment Attributions Identifying Semantic Connections Underlying Free-Text Attributions The first dimension ranged from primarily biomechanical words to primarily mind- or brain-related words. The location of each participant’s posttreatment attributions in the first dimension is shown, with 95% CIs around each condition’s mean location (top). Participants randomized to PRT vs placebo and usual care were significantly further toward the mind and brain end of the first dimension, and scores further toward the mind and brain end of the first dimension were associated with greater pain reduction.

The first principal component identified by the data-driven text scaling algorithm ranged from predominantly structural or mechanical words to predominantly mind and brain words (eg, from *car* and *scoliosis* to *anxiety* and *stress*) (Figure 4). Posttreatment locations in this semantic dimension exhibited large group differences (PRT vs placebo: *Z* = 4.55; *P* < .001; *g* = 1.03 [95% CI, 0.59-1.48]; PRT vs usual care: *Z* = 4.57; *P* < .001; *g* = 1.02 [95% CI, 0.59-1.46]). Within the PRT condition, posttreatment semantic location further toward the mind and brain direction was significantly associated with decreases in pain intensity (standardized β = −0.32, t_127_ = −2.35; *P* = .02), consistent with hypotheses.

#### Automated Attribution Scoring Algorithm

Agreement between the automated word-counting algorithm and the human coder–derived scores was substantial at posttreatment (Cohen κ = 0.68 [95% CI, 0.52-0.84]; *Z* = 8.38; *P* < .001), and moderate at pretreatment (Cohen κ = 0.42 [95% CI, 0.17-0.67]; *Z* = 3.27, *P* = .001). Examination of confusion matrices revealed that disagreement was driven primarily by the automated algorithm considering attributions as mind- or brain-related when human coders did not. For example, *childhood injury* was miscategorized as mind and brain–related by the automated algorithm due to the presence of the word *childhood* in the expert-derived list.

## Discussion

We investigated how participants think about the underlying causes of their chronic back pain in their own words, and we tested how changes in pain attributions support pain reductions in pain reprocessing therapy (PRT). At baseline, few attributions pertained to mind or brain processes, even though many to most cases of chronic pain have a centralized component.^1,2,3^ Relative to control conditions, PRT led to large increases in mind- and brain-related attributions, demonstrating target engagement. Increases in mind and brain attributions were associated with reductions in pain intensity at posttreatment and mediated the effects of PRT on 1-year follow-up pain intensity, consistent with hypothesized mechanisms of PRT.

Reattribution of pain from body to brain may be a valuable therapeutic target that is not a focus in leading psychological treatments (eg, cognitive behavioral therapy, acceptance and commitment therapy). These treatments typically present the causes of pain as complex or unknowable and describe the brain as providing “gate control,” a metaphor suggesting modulation of afferent nociceptive input. In contrast, PRT and related treatments (eg, Emotional Awareness and Expression Therapy, Explain Pain, and others^29,30,31,32^) provide the explicit affirmation that primary (nociplastic) pain is generated primarily by mind or brain processes.

A theoretical focus on attributions is consistent with active inference and predictive processing models of brain function. In these models, the brain integrates prior beliefs and incoming sensory data to update generative models of the sources of sensations.^33,34,35,36^ A shift in the generative model—such that pain is attributed to central neuroplasticity, not peripheral injury—can change how the brain prioritizes, categorizes, and constructs the sensation of pain, directly changing the pain experience.^37^

An emphasis of reattribution in PRT is that mind- or brain-generated pain is nondangerous. This emphasis is supported by our finding that increased mind and brain attribution was associated with reduced harm beliefs and activity avoidance. (Although, surprisingly, greater mind-brain attributions were associated with greater pain intensity at pretreatment—see eAppendix in Supplement 2.) At the biological level, fear reduction engages both prefrontal and amygdala pathways, 2 structures known to regulate pain in part by projections to the brainstem and spinal cord.^38,39,40,41^ Prior functional magnetic resonance imaging analyses from this trial found that PRT vs control altered prefrontal and somatosensory function.^6^ A likely function of some prefrontal-somatosensory pathways includes inferring (modeling) the causes of sensory input, and the neurobiological changes we observed may reflect the attribution changes described here.

Attribution words related to emotions (eg, *anxiety*, *fear*, *feelings*) increased in the PRT condition. Reattribution is not just a so-called “cold” cognitive process but may be integrated with other emotion-focused changes happening in PRT.^42^ Attributing pain to emotions may also motivate patients to address long-standing emotional issues or difficult relationships, as in other treatment approaches.^32,43,44^

Natural language methods complement traditional self-reported rating scales.^11,45,46^ These methods provide an open-ended format less constrained by researchers’ hypotheses and perhaps more closely capturing patients’ spontaneous beliefs, which may more closely govern spontaneous behavior. These methods also provide quantitative outputs and can be scaled. For example, the automated mind-brain scoring algorithm developed here may have fruitful applications to existing large text corpuses (eg, electronic health record data, online patient discussion forums), allowing automated measurement of symptom attributions across a range of contexts. Automated or scalable methods could facilitate the study of how attributions differ across pain conditions or across cultures in large samples, although further algorithm validation (eg, by comparison with human coder categorization) will be needed.

### Limitations

This study had several limitations, including the modest amount of variance in pain reduction explained by pain reattribution (approximately 9%). As some participants had large attribution changes with no change in pain (Figure 3B), reattribution alone is not sufficient for pain relief. The automated algorithm score agreement at pretreatment was only moderate, suggesting that further refinement is needed especially in untreated populations. Additionally, our sample was predominantly White, well-educated, and recruited from a single metropolitan area; future studies must sample more diverse populations.

## Conclusions

In this secondary analysis of a randomized trial of PRT, the reattribution of primary CBP to mind- or brain-related causes was associated with reductions in pain intensity, with modest effect sizes. While the influence of several pain beliefs on chronic pain is well recognized (eg, pain catastrophizing, pain acceptance), patients’ causal symptom attributions have been understudied. Pain attributions will guide major treatment decisions (eg, surgery vs psychotherapy) and are central in emerging neuroscientific models of brain function. Patients’ attributions of chronic pain to tissue damage are often inaccurate, and therapeutic reattribution to brain processes can support recovery from chronic pain.

## Notes

Supplement 1.
Trial Protocol

Supplement 2.
eMethods.

eResults.
**eAppendix.** Supplemental Discussion **eTable 1.** Number of Attributions in Each Category for Each Group at Each Timepoint **eTable 2.** Words With Largest Pre-to-Posttreatment Changes in Frequency Among Participants Randomized to PRT, Derived From Participants’ Attributions Regarding Causes of Pain **eFigure.** Word Clouds Showing Common Words Used in Participants’ Pain Attributions

Supplement 3.
Data Sharing Statement
