# Supplementary material for: Reattribution to Mind-Brain Processes and Recovery From Chronic Back Pain: A Secondary Analysis of a Randomized Clinical Trial
Source: JAMA Netw Open. 2023 Sep 28;6(9):e2333846. doi: 10.1001/jamanetworkopen.2023.33846 (PMC10539987; doi:10.1001/jamanetworkopen.2023.33846)
Supplement: Supplement 2. — eMethods. eResults. eAppendix. Supplemental Discussion eTable 1. Number of Attributions in Each Category for Each Group at Each Timepoint eTable 2. Words With Largest Pre-to-Posttreatment Changes in Frequency Among Participants Randomized to PRT, Derived From Participants’ Attributions Regarding Causes of Pain eFigure. Word Clouds Showing Common Words Used in Participants’ Pain Attributions [file jamanetwopen-e2333846-s002.pdf]

Supplemental Online Content

Ashar YK, Lumley MA, Perlis RH, Liston C, Gunning FM, Wager TD. Reattribution to mind-brain processes and recovery from chronic back pain. *JAMA Netw Open.* 2023;6(9):e2333846. doi:10.1001/jamanetworkopen.2023.33846

eMethods.

eResults.

eAppendix. Supplemental Discussion

eTable 1. Number of Attributions in Each Category for Each Group at Each Timepoint

eTable 2. Words With Largest Pre-to-Posttreatment Changes in Frequency Among Participants Randomized to PRT, Derived From Participants' Attributions Regarding Causes of Pain

eFigure. Word Clouds Showing Common Words Used in Participants' Pain Attributions

This supplemental material has been provided by the authors to give readers additional information about their work.

© 2023 Ashar YK et al. *JAMA Network Open.*

eMethods.

Sample size and power analyses. Power analysis determined sample size using a meta-analytic estimate of Cohen's *d* = .62 for CBP pain intensity for psychological treatment vs. treatment-as-usual.[21] Effects of this size require n = 43 per group to achieve 80% power at α = .05. We aimed to enroll 50 per group, accounting for anticipated attrition, with n = 50 or 51 patients ultimately randomized to each group.

Pain reprocessing therapy (PRT). Participants completed a 1-hour telehealth session with a physician assessing centralized versus peripheral contributions to pain and providing education on mind and brain generators of chronic pain. The diagnosis of centralized pain was substantiated by personalized supporting evidence (e.g., spatial spread of symptoms, history of multiple somatic symptoms).[18] Participants then received 8 individual 1-hr therapy sessions twice weekly for 4 weeks. Main PRT techniques include a) providing personalized evidence that pain is due to mind/brain processes; b) somatic-focused exercises guiding the reattribution of pain sensations to non-dangerous, brain-generated processes; and c) engagement in feared, pain-associated activities. Adjunctive techniques include d) addressing psychosocial threats potentially amplifying pain (e.g., difficult emotions, stress), and e) increasing positive emotions and self-compassion. See REF[6] and REF[19] for a further description of PRT.

Correlation between baseline mind/brain attribution scores and other baseline metrics. We tested how pre-treatment mind/brain attribution scores were related to validated questionnaire measures of pain beliefs at baseline. We hypothesized that mind/brain attribution scores would be a) inversely correlated with harm beliefs/activity avoidance (TSK-11) and pain catastrophizing (PCS), given that structural injury beliefs are hypothesized to promote fear and catastrophizing, and b) positively correlated with the perceived influence of stress and emotion on pain (SOPA-emotions). Exploratory analyses further tested the association between pre-treatment mind/brain attribution scores and pain intensity, age, gender, and duration of pain at pre-treatment. Spearman rank correlations were computed to account for non-normal distributions of mind/brain attribution scores.[29]

Effects of PRT on mind/brain attribution scores. To test the effect of PRT on pain attributions, we fit a general linear model (MATLAB function *fitglm*) predicting differences between conditions in pre-to-post-treatment change in mind/brain attribution scores, based on two condition x time interactions (PRT vs. placebo *x* time, PRT vs. usual care *x* time), with age and gender as covariates. For interpretability of effect sizes, we additionally computed hedge's *g* for PRT vs. placebo and PRT vs. usual care differences in pre-to-post-treatment changes in

© 2023 Ashar YK et al. *JAMA Network Open.*

mind/brain attribution scores. Exploratory tests of the placebo vs. usual care comparison were conducted as well, testing whether the open-label placebo intervention affected pain attributions.

Association between reattribution and changes in pain intensity. To test pain reattribution as a potential mechanism of PRT, we fit a general linear model (MATLAB function *fitglm*) predicting pre-to-post-treatment change in pain intensity from pre-to-post-treatment change in mind/brain attribution scores, with a condition x change in mind/brain attribution scores interaction term and covariates for age and gender. Condition was coded as a categorical variable with the PRT condition serving as the reference, so the change in attributions term tested for an effect within the PRT condition (i.e., condition variable set to zero) and the interaction term tested whether the relationship depended on condition. For enhanced interpretability of effect sizes, we also computed the simple correlation between pre-to-post-treatment changes in pain and mind/brain attribution scores across all subjects and within the PRT condition alone. To examine longer-term effects of reattribution, the above was repeated but with pain intensity at 1 year follow-up (and change from baseline to 1 year).

Association between reattribution and changes in harm beliefs and activity avoidance. To test the hypothesis that reattribution supports fear reduction, we fit a general linear model as described in the section above but with pre-to-post-treatment change in TSK-11 scores as the outcome, rather than changes in pain intensity.

Mediation analyses. A longitudinal mediation model tested whether the long-term effects of PRT on pain intensity were mediated by attribution changes. The independent variable was PRT vs. control conditions (indicator variable). Participants from both control conditions were collapsed for this model as our main results demonstrated that both controls conditions had little to no change in attributions and both controls had similar pain intensity levels at follow-up. The mediator variable was pre-to-post-treatment change in mind/brain attribution scores, and the outcome variable was change in pain intensity from pre-treatment to 1-year follow-up, with age and gender were included as covariates, as in the models described above. Significance testing was conducted via bootstrapping procedures (10,000 iterations) using the Canlab Mediation Toolbox (https://github.com/canlab/MediationToolbox).

Text scaling analyses. We submitted post-treatment data from all participants to the text-scaling analysis, owing to the low presence of mind/brain attributions in the pre-treatment data. We tested for PRT vs. placebo and PRT vs. usual care differences in participants' post-treatment location in the first semantic dimension identified by the text-scaling algorithm, using Wilcoxon rank sum test to account for non-normality and estimating effect sizes of differences

© 2023 Ashar YK et al. *JAMA Network Open.*

with hedge's *g*. Exploratory tests of placebo vs. usual care differences were conducted for archival purposes. To test reattribution as a mechanism of pain reduction in PRT, we conducted a GLM predicting pre-to-post-treatment changes in pain intensity from post-treatment location in the first semantic dimension identified by the text-scaling algorithm, with covariates for condition, age, gender, and an interaction term for condition x semantic location.

Automated Attribution Scoring Algorithm. Experts were asked to provide unigrams or bigrams (i.e., single words and 2-word combinations) of potential mind/brain attributions. Expert-derived keywords were manually expanded by the first author using a thesaurus to include synonyms. The expert-derived list and the participant attributions were preprocessed by the correction of typographical errors, lemmatization, removal of stop words ("the", "and"), and exclusion of words shorter than 2 or longer than 20 characters using the MATLAB Text Analytics toolbox.

To benchmark algorithm performance, we computed Cohen's kappa relative to the human coder-derived mind/brain attribution scores. This metric estimates the agreement between scores controlling for agreement expected by chance. Kappa was computed separately for pre- and post-treatment data, owing to the relatively low prevalence of mind/brain attributions at pre-treatment.

© 2023 Ashar YK et al. *JAMA Network Open.*

eResults

Increases in mind/brain attribution scores were associated with decreases in pain catastrophizing in the PRT condition, standardized β = -0.29, $t(127) = -2.16$, $p = .03$, controlling for covariates, consistent with hypotheses; interactions for condition x change in mind/brain attribution scores interactions were not significant. Examining simple correlations, pre-to-post-treatment changes in mind/brain attribution scores and harm beliefs/activity avoidance (TSK-11) were correlated at $r(133) = -.38$, $p < .001$ in the full sample and at $r(42) = -.35$, $p = .02$ within the PRT condition.

© 2023 Ashar YK et al. *JAMA Network Open.*

eAppendix. Supplemental Discussion

We found (unexpectedly) that pre-treatment mind/brain attribution scores were positively correlated with pain intensity. Further, higher pre-treatment mind/brain attributions were correlated with a stronger perceived influence of stress and emotion on pain (SOPA scale). It may be that participants with more intense pain had tried and failed a greater number of biomedically-oriented treatments, leading them to recognize a centralized component to their pain. Similarly, considering that this was a non-specific chronic back pain population excluding participants with fractures and tumors, it may be that more severe pain is in fact more likely to have a greater centralized component.

These findings demonstrate that having mind- or brain-related attributions is not by itself sufficient to relieve pain. It may be necessary for these attributions to be accompanied by a set of skills, tools, or other beliefs for mind-brain attributions to be helpful. For example, these may include psychological skills for processing difficult emotions or for conducting behavioral exposures, or an understanding of nociplastic pain as a “false alarm”, an understanding which may not necessarily follow from attribution alone.

© 2023 Ashar YK et al. *JAMA Network Open.*

eTable 1. Number of Attributions in Each Category for Each Group at Each Timepoint

Percentages are displayed in main manuscript Figure 2.

Attribution category	PRT Pre	PRT Post	Placebo Pre	Placebo Post	Usual Care Pre	Usual Care Post
psych	1	47	5	5	4	2
brain	1	9	0	0	0	1
stress	9	15	11	8	10	6
genetic	11	4	4	4	12	10
spinal	14	4	9	6	9	14
neglect	5	4	3	1	7	9
other	9	4	9	11	8	9
physio	23	11	27	19	21	23
age	11	2	6	7	5	3
sedentary	10	2	9	9	4	6
injury	25	16	22	31	38	31
activity	31	14	48	31	32	27

© 2023 Ashar YK et al. *JAMA Network Open.*

eTable 2. Words With Largest Pre-to-Post–Treatment Changes in Frequency Among Participants Randomized to PRT, Derived From Participants' Attributions Regarding Causes of Pain

Ngram	Example	Pre-treatment frequency	Post-treatment frequency	Change in frequency
Decreases in frequency				
activity	'activity'	18	4	-14
posture	'poor posture'	27	18	-9
sport	'sports injuries'	16	8	-8
weight	'extra weight'	14	6	-8
disc	'herniated disc'	8	0	-8
age	'aging'	17	10	-7
heavy	'heavy lifting'	7	0	-7
fall	'falling – ice skating as a child'	9	3	-6
genetics	'genetics'	13	7	-6
spine	'curved spine'	6	0	-6
work	'sedentary work'	15	9	-6
physical	'physical movements'	12	7	-5
related	'sports related injury'	5	0	-5
back	'back alignment'	11	7	-4
bad	'bad aftercare'	10	6	-4
break	'broken bone'	4	0	-4
cause	'over working my back muscles to cause spinal disc to bulge'	6	2	-4
degenerative	'degenerative disc disease'	4	0	-4
desk	'sitting at a desk job'	4	0	-4
Increases in frequency				
anxiety	'anxiety'	3	13	10
pathway	'brain pathways that developed and stayed even after healing'	0	8	8

© 2023 Ashar YK et al. *JAMA Network Open.*

fear	‘fear of injury’	0	7	7
neural	‘neural pathways’	0	6	6
day	‘sitting most of the day’	0	4	4
factor	‘psychological factors’	0	4	4
feel	‘not paying attention to my feelings’	0	4	4
structural	'perception of potential structural damage'	0	4	4
emotion	‘repressed emotions’	0	3	3
need	'putting everyone else's needs before my own'	0	3	3
people	'culture of pain and many people showing pain around me'	0	3	3
structural	'perception of potential structural damage'	1	4	3
tense	'feeling tense; physically tensing up my body'	0	3	3

© 2023 Ashar YK et al. *JAMA Network Open.*

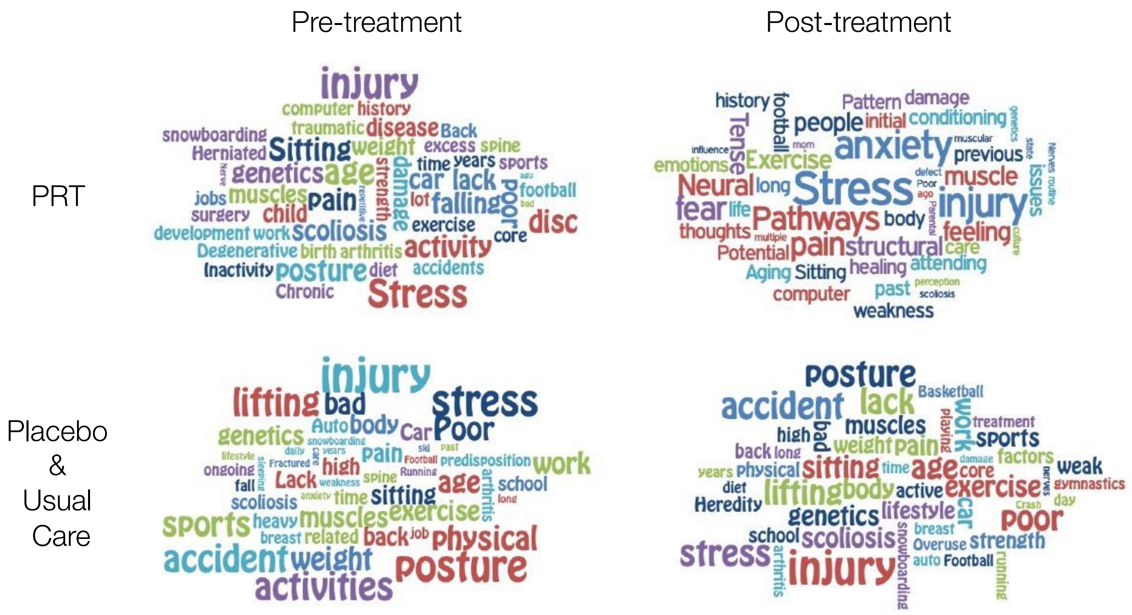

eFigure. Word Clouds Showing Common Words Used in Participants' Pain Attributions

Attributions describe participants' beliefs about the causes of their chronic back pain. Font size scales with word frequency. Data from the placebo and usual care control conditions were similar and are shown combined for ease of visualization. Figure created using www.edwordle.net.

© 2023 Ashar YK et al. *JAMA Network Open.*
