# Supplementary material for: Reattribution to Mind-Brain Processes and Recovery From Chronic Back Pain: A Secondary Analysis of a Randomized Clinical Trial
Source: JAMA Netw Open. 2023 Sep 28;6(9):e2333846. doi: 10.1001/jamanetworkopen.2023.33846 (PMC10539987; doi:10.1001/jamanetworkopen.2023.33846)
Supplement: Supplement 3. — Data Sharing Statement [file jamanetwopen-e2333846-s003.pdf]

Data Sharing Statement

Ashar. Reattribution to Mind-Brain Processes and Recovery from Chronic Back Pain. *JAMA Netw Open*. Published September 28, 2023. doi:10.1001/jamanetworkopen.2023.33846

Data
Data available: Yes
Data types: Deidentified participant data, Data dictionary
How to access data: https://github.com/yonestar/Ashar_2023_CBP_reattribution
When available: With publication

Supporting Documents
Document types: None

Additional Information
Who can access the data: anyone interested in the data
Types of analyses: all
Mechanisms of data availability: publicly posted
